# N-Acylethanolamine Acid Amidase Inhibition Potentiates Morphine Analgesia and Delays the Development of Tolerance

**DOI:** 10.1007/s13311-021-01116-4

**Published:** 2021-09-22

**Authors:** Mauro Congiu, Laura Micheli, Michele Santoni, Claudia Sagheddu, Anna Lisa Muntoni, Alexandros Makriyannis, Michael S. Malamas, Carla Ghelardini, Lorenzo Di Cesare Mannelli, Marco Pistis

**Affiliations:** 1 Division of Neuroscience and Clinical Pharmacology, Department of Biomedical Sciences, University of Cagliari, Cagliari, Italy; 2 Section of Pharmacology and Toxicology, Department of Neuroscience, Psychology, Drug Research and Child Health - Neurofarba, Università Degli Studi Di Firenze, Florence, Italy; 3 Neuroscience Institute, National Research Council of Italy (CNR), Cagliari, Italy; 4 Department of Pharmaceutical Sciences, Department of Chemistry and Chemical Biology, Center for Drug Discovery, Northeastern University, Boston, MA USA

**Keywords:** *N*-acylethanolamine acid amidase, Morphine, Analgesia, Electrophysiology, Glial activation, Locus coeruleus

## Abstract

Opioids are essential drugs for pain management, although long-term use is accompanied by tolerance, necessitating dose escalation, and dependence. Pharmacological treatments that enhance opioid analgesic effects and/or attenuate the development of tolerance (with a desirable opioid-sparing effect in treating pain) are actively sought. Among them, *N*-palmitoylethanolamide (PEA), an endogenous lipid neuromodulator with anti-inflammatory and neuroprotective properties, was shown to exert anti-hyperalgesic effects and to delay the emergence of morphine tolerance. A selective augmentation in endogenous PEA levels can be achieved by inhibiting *N*-acylethanolamine acid amidase (NAAA), one of its primary hydrolyzing enzymes. This study aimed to test the hypothesis that NAAA inhibition, with the novel brain permeable NAAA inhibitor AM11095, modulates morphine’s antinociceptive effects and attenuates the development of morphine tolerance in rats. We tested this hypothesis by measuring the pain threshold to noxious mechanical stimuli and, as a neural correlate, we conducted in vivo electrophysiological recordings from pain-sensitive locus coeruleus (LC) noradrenergic neurons in anesthetized rats. AM11095 dose-dependently (3–30 mg/kg) enhanced the antinociceptive effects of morphine and delayed the development of tolerance to chronic morphine in behaving rats. Consistently, AM11095 enhanced morphine-induced attenuation of the response of LC neurons to foot-shocks and prevented the attenuation of morphine effects following chronic treatment. Behavioral and electrophysiological effects of AM11095 on chronic morphine were paralleled by a decrease in glial activation in the spinal cord, an index of opioid-induced neuroinflammation. NAAA inhibition might represent a potential novel therapeutic approach to increase the analgesic effects of opioids and delay the development of tolerance.

**Supplementary Information:**

The online version contains supplementary material available at 10.1007/s13311-021-01116-4.

## Introduction

Opioids are essential drugs for pain management, although long-term use is accompanied by tolerance, necessitating dose escalation, and dependence. In recent years, opioid prescriptions increased exponentially, especially in the USA, resulting in serious public health problems, such as addiction, overdose, and fatalities [1–3]. One of the major problems of opioid use is the development of tolerance to the analgesic effect, leading to dosage increases and to the exacerbation of drug side effects, such as constipation, addiction, and respiratory depression, which undergo different degrees of tolerance. The mechanisms that underlie the development of opioid tolerance are diverse and extensively studied and involve receptor desensitization, phosphorylation, uncoupling with intracellular effectors and recycling [4], opioid-induced glial activation, and neuroinflammation [5, 6]. Thus, it is imperative to investigate pharmacological treatments that enhance opioid analgesic effects and/or attenuate the development of tolerance (with a desirable opioid-sparing effect in treating pain).

*N*-acylethanolamines (NAEs), endocannabinoid-like lipid neuromodulators such as palmitoylethanolamide (PEA), show promise in treating inflammation and pain. Among NAEs, the most studied in the context of pain is PEA. In fact, PEA was characterized as an anti-inflammatory, neuroprotective, and anti-hyperalgesic medication [7–10]. Tissue levels of PEA are increased in brain areas involved in nociception and in the spinal cord following neuropathic pain induction and in human conditions associated with pain [11, 12].

Anti-hyperalgesic and anti-inflammatory properties of PEA might result helpful against the development of opioid tolerance. The efficacy of combination therapy of opioids with PEA has been successfully explored in recent preclinical behavioral investigations [13, 14]. It has been shown that PEA potentiates the antinociceptive effects of morphine and delays the development of tolerance in rats [13]. Importantly, this study also revealed that PEA prevented astrocyte activation in the dorsal horn. Similarly, the same strategy proved effective in a rat model of oxaliplatin-induced neuropathy [15].

The bioavailability of PEA is relatively low due to its lipophilic nature and sensitivity to hydrolyzing enzymes. Thus, we sought alternative pharmacological approaches to augment endogenous PEA tissue levels. One strategy can be pursued with indirect agonists, such as *N*-acylethanolamine acid amidase (NAAA) inhibitors [16–18]. NAAA is the lysosomal cysteine hydrolase mainly involved in PEA degradation [19] and is a target for small-molecule inhibitors to increase tissue levels of PEA [18, 20, 21]. Therefore, we utilized a highly potent and selective inhibitor of human NAAA, AM11095 (IC_50_ = 20 nM) and tested its ability to potentiate morphine’s antinociceptive properties and delay tolerance in rats. We also explored the ability of AM11095 to modulate morphine’s effects on the response of locus coeruleus (LC) noradrenergic (NA) neurons to noxious stimuli, a neural correlate of its antinociceptive effects, and on the tolerance that develops after chronic morphine administrations. Additionally, glial activation in the spinal cord, as an index of opioid-induced neuroinflammation, was studied following chronic morphine administration.

We report that the NAAA inhibitor AM11095 enhanced morphine-induced antinociception, delayed tolerance development, and reduced chronic morphine-induced glial activation in the spinal cord.

## Methods

### Drugs

AM11095 was designed and synthesized at the Center for Drug Discovery, Northeastern University, as described in US Patent 9,963,444 B2, 2018 and [22]. AM11095 is a slowly reversible NAAA inhibitor with a half-maximal inhibitory concentration (IC_50_) value of 20 nM, while having no effect on serine hydrolases FAAH and MGL activity at concentrations > 10 μM [22]. A detailed pharmacokinetics analysis of AM11095 (compound 36) in mice is described by Malamas et al. [22]. AM11095 was tested in CEREP “off-target” panels consisting of receptors (i.e., 5HT1A, 5HT2A, 5HT2C, DA D1, DA D2, DA D3, DA D4, delta-opioid, κ-opioid, and μ-opioid) and transporters (i.e., DAT, SERT, and NET) and did not show any effect at 10 µM [22]. AM11095 was dissolved in tween80, ethanol, and saline (1:1:18) when injected intraperitoneally (i.p.) or 1% carboxymethylcellulose sodium salt (CMC) when administered p.o. Morphine (S.A.L.A.R.S., Como, Italy) was dissolved in saline.

### Animals and Treatments

Male Sprague–Dawley (Envigo, Varese, Italy) rats weighing 200–250 g were group-housed and kept on a regular 12:12-h light/dark cycle, in temperature- and humidity-controlled facilities, with food and water available ad libitum. Rats were left to acclimatize at least 1 week after their arrival. All animal manipulations were carried out according to the Directive 2010/63/EU of the European Parliament and of the European Union council (22 September 2010) on the protection of animals used for scientific purposes. The ethical policy of the Universities of Florence and Cagliari complies with the Guide for the Care and Use of Laboratory Animals of the US National Institutes of Health (NIH Publication No. 85–23, revised 1996; University of Florence assurance number: A5278-01). Formal approval to conduct the experiments described was obtained from the Italian Ministry of Health (No. 498/2017-PR) and from the Animal Subjects Review Board of the University of Florence and the Animal Ethics Committees of the University of Cagliari. Experiments involving animals have been reported according to ARRIVE guidelines [23]. The experimental protocols were conducted to minimize pain and suffering and to reduce the number of animals used.

For behavioral and ex vivo experiments, AM11095 was administered p.o. at doses of 3, 10, and 30 mg/kg daily (split into two daily treatments of 1.5, 5, and 15 mg/kg performed once in the morning and once in the evening). AM11095 was previously studied in a full battery of functional observational battery (FOB) tests [18] at doses up to 25 mg/kg i.p. to assess any effect on behavior, locomotor activity, autonomic reflexes, muscle tone, and others. Results showed the lack of any effect [18]. Additionally, AM11095 does not show any effect on conditioned place preference as an index of potential abuse liability [18].

Treatments started on day −8 as pretreatment and continued during the following days until the end of the experiments. On day 1, morphine was dissolved in saline and daily subcutaneously (s.c.) injected at the dose of 10 mg/kg. Analgesia measurements were conducted daily before and 30 min after the acute administration of morphine and before the first AM11095 daily treatment by the paw pressure test.

In the other two experiments regarding morphine tolerance, AM11095 (15 mg/kg) was also administered daily p.o. and i.p. starting on day −8 until the end of the experiments. Control animals received an equal volume of vehicle. After the behavioral test, tissues were collected as follows. Animals were sacrificed by decapitation, and the lumbar spinal cord was collected and fixed by immersion in 4% neutral buffered formalin. Morphine-treated rats were sacrificed when tolerance was established (day 5), while morphine + AM11095 animals were split up into two groups, one sacrificed on day 5 and the other on day 12, when morphine tolerance was not or was settled respectively. Control animals were sacrificed at the end of the experiments (day 12).

For electrophysiological experiments, AM11095 was dissolved in tween80, ethanol, and saline (1:1:18) when injected i.p. for acute studies or 1% carboxymethylcellulose sodium salt (CMC) when administered p.o. Morphine was dissolved in saline. AM11095 (15 mg/kg i.p.) was administered 30 min before morphine (1 mg/kg i.v.) injection in acute experiments. For the experiments on morphine tolerance, each rat received AM11095 (15 mg/kg p.o.) or its vehicle once per day for 13 days, including the day of the electrophysiological recording; moreover, 30 min after AM11095 administration, each rat received an injection of morphine (10 mg/kg s.c.) for 6 days before the electrophysiological recording.

### Nociceptive Threshold Analysis by Paw Pressure Test

The nociceptive threshold in the rat was determined with an analgesimeter (Ugo Basile, Varese, Italy) according to the method described by Leighton et al. [24]. Briefly, a constantly increasing pressure was applied to a small area of the dorsal surface of the hind paw using a blunt conical mechanical probe. Mechanical pressure was increased until vocalization or a withdrawal reflex occurred while rats were lightly restrained. Vocalization or withdrawal reflex thresholds were expressed in grams. These limits assured a more precise determination of mechanical withdrawal threshold in experiments aimed to determine the effect of treatments. An arbitrary cut-off value of 250 g was adopted in order to avoid damage to the paw. The data were collected by an observer who was blinded to the protocol.

### In Vivo Single-Unit Extracellular Recordings

Rats were anesthetized with urethane (1.3 g/kg, i.p.) and, for intravenous (i.v.) administration of pharmacological agents, a cannula was inserted into their femoral vein. Rats were placed in a stereotaxic apparatus (Kopf, Tujunga, CA, USA) with their body temperature maintained at 37 ± 1 °C by a heating pad. The recording electrode (impedance 5.0–7.0 MΩ) was placed at these coordinates according to the stereotaxic rat brain atlas of Paxinos and Watson [25]: AP, − 10 ± 0.5 mm from the bregma; L, 1.3 ± 0.2 mm from the midline; V, 5.5–6.5 mm from the cerebellum cortical surface. Putative NA neurons were isolated and identified according to already published criteria [26] such as (i) a broad (3–4 ms in duration), often notched, biphasic waveform; (ii) slow spontaneous discharge (0.5–6.0 Hz); (iii) a typical response to noxious stimuli such as contralateral foot or tail pinch by an increase in activity followed by a quiescent interval; and (iv) the inhibition by the α2-adrenoceptor agonist clonidine. Bursts were defined as the occurrence of two spikes at interspike interval < 80 ms and terminated when the interspike interval exceeded 160 ms. Single-unit activity of neurons was recorded extracellularly (bandpass filter 0.1–10,000 Hz) with glass micropipettes filled with 2% Pontamine sky blue (PSB) dissolved in 0.5 M sodium acetate. Individual action potentials were isolated and amplified employing a window discriminator (Neurolog System, Digitimer, Hertfordshire, UK), and displayed on a digital storage oscilloscope (TDS 3012, Tektronics, Marlow, UK). Experiments were sampled online and offline with Spike2 software by a computer connected to CED1401 interface (Cambridge Electronic Design, Cambridge, UK).

A total of 50 rats were used and divided into 4 groups: vehicle i.p. + morphine (1 mg/kg i.v.), AM11095 (15 mg/kg i.p.) + morphine (1 mg/kg i.v.), AM11095 (15 mg/kg p.o.) + morphine (10 mg/kg s.c.) + morphine (1 mg/kg i.v.), vehicle p.o. + morphine (10 mg/kg s.c.) + morphine (1 mg/kg i.v.). The basal activity was recorded for at least 3 min. Electrical pulses were generated by a stimulator (Digitimer, DS3 model) and they were applied using bipolar needle electrodes (26-gauge, 2-mm separation) inserted subcutaneously into the medial-external surface of the left hind paw corresponding to the zone of innervation by the sciatic nerve and contralateral to the LC being recorded. Foot-shock stimuli (5.0 ms in duration, 10 mA in intensity) were delivered every 2 s with a total of 50 repetitions per train.

At the end of recording sessions, DC current (15 mA for 15 min) was passed through the recording micropipette in order to eject PSB for marking the recording site. Brains were then rapidly removed and were frozen in isopentane cooled to −40 °C. The position of the electrodes was microscopically identified on serial 60 μm sections stained with Neutral Red.

### Immunohistochemistry of the Spinal Cord

Formalin-fixed cryostat 7-μm sections of the spinal cord were incubated for 1 h in blocking solution (Bio-Optica; Italy) at room temperature; then sections were incubated for 24 h at 4 °C in PBST containing primary antisera and 5% normal donkey serum. The primary antibody was directed against Iba1 (rabbit antiserum, 1:500; Wako Chemicals, USA; [27]) for microglial staining and against the glial fibrillary acidic protein (GFAP; rabbit antiserum, 1:500; Dako, USA; [28]) for astrocyte staining. The following day, slides were washed 3 × with PBS and then sections were incubated in the dark with secondary antibody, goat anti-rabbit IgG labeled with Alexa Fluor 568 (1:500), in PBST at room temperature for 2 h. After 3 × PBS 0.3% Triton X-100 wash for 10 min, the sections were incubated with DAPI, a nuclei marker, at room temperature for 5 min and then the slides were mounted using Fluoromount™ (Life Technologies-Thermo Scientific, Rockford, IL, USA) as a mounting medium. For all immunohistochemical studies, negative control sections (no exposure to the primary antisera) were processed concurrently with the other sections.

Negative control sections (no exposure to the primary antisera) were processed concurrently with the other sections for all immunohistochemical studies. Images were acquired using a motorized Leica DM6000 B microscope equipped with a DFC350FX camera (Leica, Mannheim, Germany).

The mean fluorescence intensity of GFAP was calculated by subtracting the background (multiplied by the total area) from the GFAP integrated intensity. The GFAP signal in immunostained sections was quantified using ImageJ software (NIH, Bethesda, MD, USA) by automatic thresholding images with the aid of the “triangles” algorithm, which we found to provide the most consistent pattern recognition across all acquired images. Analyses were performed on three different images for each animal, collected through a 20 × objective.

Quantitative analysis of GFAP and Iba1-positive cells was performed by collecting at least three independent fields through a 20 × 0.5NA objective. GFAP-positive cells were counted using the “cell counter” plugin of ImageJ, while Iba1-positive cells were quantified by means of the automatic thresholding and segmentation features of ImageJ.

Morphological analysis of astrocytes was performed using the plugin neurite tracker by ImageJ. Five astrocytes for each animal collected through a 40 × objective were analyzed and the following parameters were considered: total number of processes, number of primary and secondary processes, total process length, primary and secondary process length, average of primary and secondary process length, number of total connections.

### Statistical Analysis

For in vivo experiments on morphine tolerance, behavioral measurements were performed on five rats for each treatment carried out in two different experimental sets. Results were expressed as mean ± (SEM) with one-way analysis of variance. A Bonferroni’s significant difference procedure was used as a post hoc comparison. *p*-values < 0.05 were considered significant. Data were analyzed using the Origin 9 software (OriginLab, Northampton, MA, USA).

For in vivo electrophysiology, drug-induced changes in firing rate and regularity were calculated by averaging the effects of the drugs for the 2-min period following drug administration and comparing them to the mean of the pre-drug baseline. Changes in neuron response to foot-shock after morphine injection were calculated as the number of spikes evoked in the specific time window expressed in percentage compared with the spikes evoked during basal. All the numerical data are given as mean ± SEM. Statistical significance was assessed using two-way ANOVA for repeated measures, Student’s *t*-test, or Mann–Whitney test when appropriate. Post hoc multiple comparisons were made using Bonferroni’s test. In all cases, *p* < 0.05 was considered significant and determined using the calculation software GraphPad Prism.

## Results

### AM11095 Acutely Enhanced Morphine Analgesic Effects

**Fig. 1:**
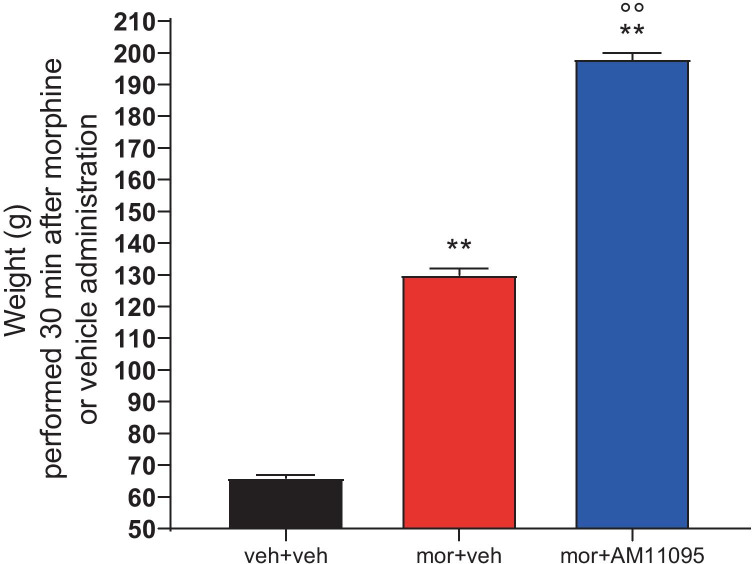
Potentiation of acute analgesic action of morphine by AM11095. AM11095 (30 mg/kg administered for 8 days) and morphine (10 mg/kg s.c.) were injected on day 8. The nociceptive threshold was measured before (0 min) and 30 min after by the paw pressure test. Control animals were treated with vehicles. Data are expressed as mean ± SEM of value from 5 rats analyzed in two different experimental sets. **p < 0.01 vs vehicle + vehicle; °°*p* < 0.01 vs morphine + vehicle

Previous studies demonstrated that PEA is able to potentiate morphine antinociceptive properties [13, 14]. Therefore, we investigated whether NAAA inhibitor AM11095 could have a similar effect by indirectly augmenting tissue PEA levels. We carried out the paw pressure test in order to measure nociceptive thresholds in three groups of rats receiving either vehicle, morphine s.c. 10 mg/kg and vehicle, or morphine s.c. 10 mg/kg and AM11095 30 mg/kg per o.s. for 8 days. The test was performed 30 min after drug administration. As expected, morphine exerted its antinociceptive effect by increasing the nociceptive threshold (Fig. 1). Importantly, AM11095 co-administration further increased the weight that rats could tolerate (Fig. 1), suggesting that NAAA inhibition potentiated morphine antinociceptive effects.

### AM11095 Acutely Increased Morphine Effects on Foot-Shock-Evoked Responses

**Fig. 2:**
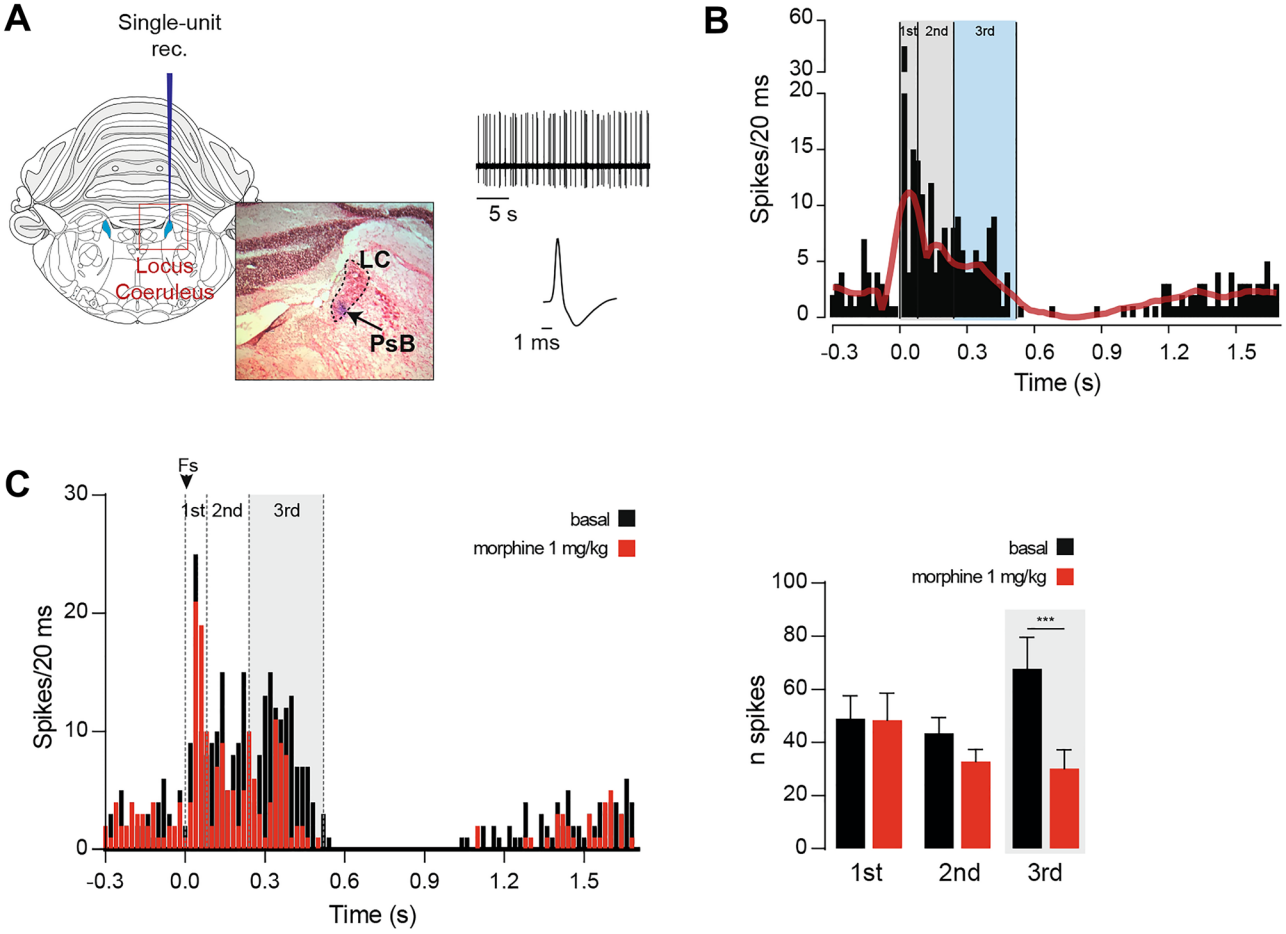
Morphine attenuates the response of locus coeruleus (LC) neurons to foot-shock. (**A**) Left: coronal brain section highlighting the LC and one histological localization example of the recording site. Right: representative electrophysiological trace of a LC neuron and its typical action potential waveform. (**B**) Example of a peristimulus time histogram (PSTH) of a LC neuron responding to a set of FS (5 ms, 10 mA, 0.5 Hz); the red overlaid line represents the smoothed average of 19 neurons. (**C**) Left: representative PSTH of a neuron responding to the FS protocol before and after i.v. injection of morphine (1 mg/kg). Right: bar graph representing the quantification of the number spikes before and after morphine injection in the three different phases (1st phase 0–80 ms, 2nd phase 80–240 ms, 3rd phase 240–520 ms). Data are expressed as mean ± SEM. *** *p* < 0.0001

Noradrenergic (NA) locus coeruleus (LC) is a key area contributing to the development of morphine dependence, tolerance, and withdrawal. LC represents the ideal candidate to study AM11095 effects on morphine properties since its neurons respond to nociceptive stimuli and are sensitive to opioids [29, 30]. Therefore, we performed in vivo single-unit extracellular recordings of LC NA neurons in anesthetized rats (Fig. 2a). We assessed their response to nociceptive stimuli (Fig. 2b), i.e., 50 foot-shocks (FS; 0.5 Hz, 10 mA, 5 ms), then the effect of i.v. morphine (1 mg/kg) on the response to FS. We identified LC NA neurons by their well-defined electrophysiological features (Fig. 2a) and delivered a set of 50 FS to the contralateral paw. As expected, LC NA neurons respond to FS in a triphasic fashion [29] (Fig. 2b). After the first set of FS, we injected a single dose of morphine and repeated the FS protocol. In line with previous studies, we found that morphine effects were specific to the third phase of the FS response (Fig. 2c; two-way ANOVA, morphine effect: *p* < 0.0001; Bonferroni’s tests 1st before 48.9 ± 8.7 vs 1st after 48.3 ± 10.2: *t*_24_ = 0.10, *p* > 0.999; 2nd before 43.4 ± 5.0 vs 2nd after 32.8 ± 4.5: *t*_24_ = 1.94, *p* = 0.193; 3rd before 67.8 ± 11.7 vs 3rd after 30.2 ± 7.0: *t*_24_ = 6.83, *p* < 0.0001; *n* = 9), a neural correlate of morphine antinociceptive effects [29, 30].

**Fig. 3:**
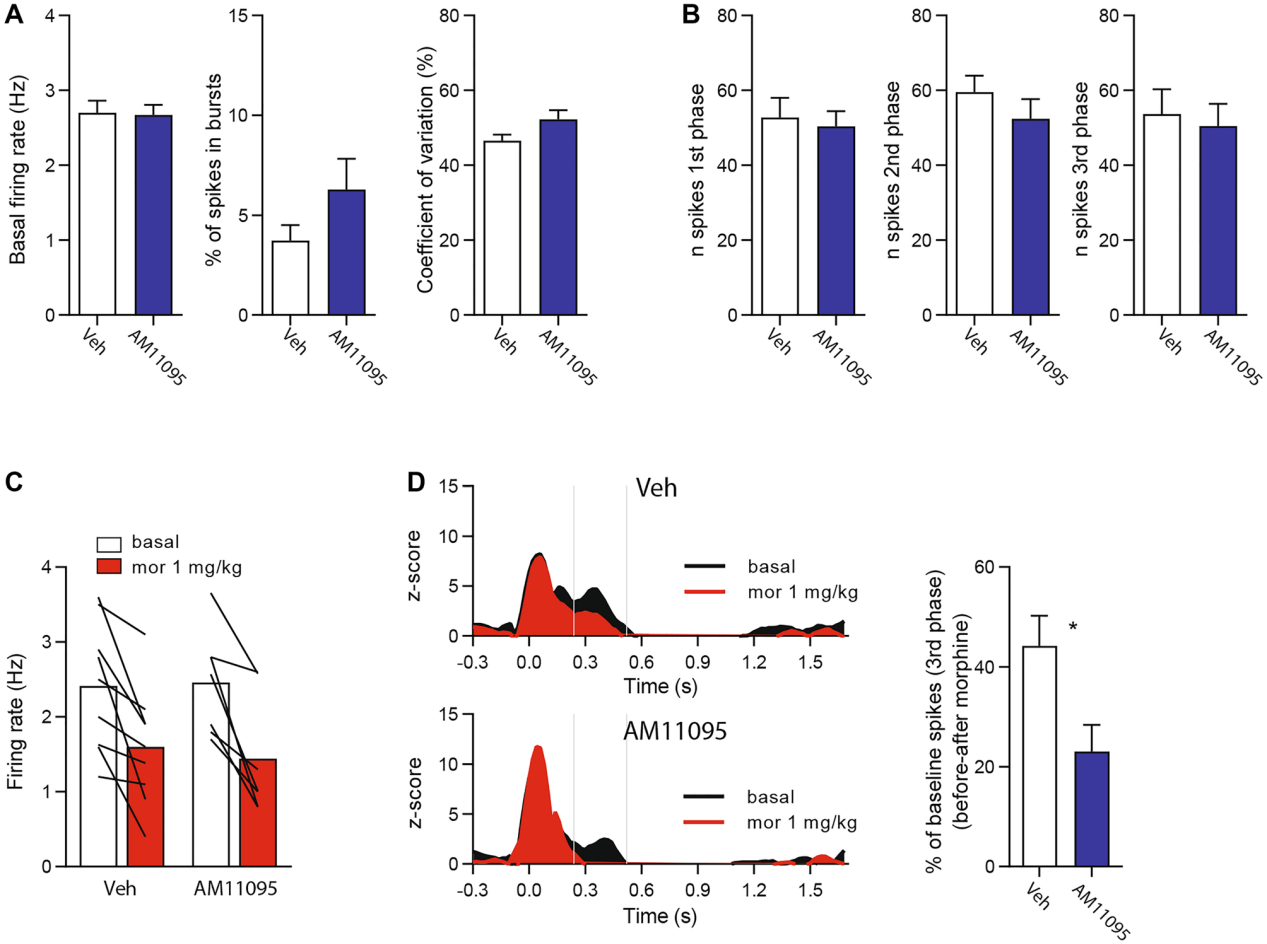
AM11095 does not change baseline electrophysiological properties of locus coeruleus (LC) neurons but potentiates morphine antinociception. (**A**) Bar graph representing the spontaneous electrophysiological properties of LC neurons recorded from vehicle treated (Veh) or AM11095-treated rats. Left: firing rate, center: burst firing, right: coefficient of variation. (**B**) Bar graph representing the response of LC neuron to FS. Left: 1st phase, center: 2nd phase, right: 3rd phase. (**C**) Bar graph representing morphine-induced reduction of the firing rate of LC neurons. (**D**) Left: average PSTH of the responses of LC neurons to foot-shock before and after morphine injection in vehicle (Veh, top) and AM11095 (bottom) pre-treated rats. Right: bar graph illustrating that AM11095 enhanced morphine-induced reduction of the response to FS in LC neurons. Data are expressed as mean ± SEM. *p < 0.05

On the basis of these observations, we treated rats with a single dose i.p. of the AM11095 (15 mg/kg, i.p.) or its vehicle, and assessed (i) basal electrophysiological properties, (ii) response to FS, and (iii) morphine effects to both firing and FS response. AM11095 acute treatment left unaltered spontaneous electrophysiological properties (Fig. 3a) such as firing rate (veh 2.7 ± 0.2 Hz vs AM11095 2.7 ± 0.1 Hz, unpaired *t* test: *t* = 0.14, *p* = 0.89, *n*_veh_ = 50, *n*_AM11095_ = 42), burst firing (veh 3.7 ± 0.8% vs AM11095 6.3 ± 1.5%, Mann–Whitney test: *U* = 939.5, *p* = 0.38, *n*_veh_ = 50, *n*_AM11095_ = 42), and coefficient of variation (veh 46.6 ± 1.6% vs AM11095 52.3 ± 2.5%, Mann–Whitney test: *U* = 841, *p* = 0.1, *n*_veh_ = 50, *n*_AM11095_ = 42), response to FS (Fig. 3b) at the 1st (veh 52.8 ± 5.2 vs AM11095 50.4 ± 4.1, unpaired *t* test: *t* = 0.37, *p* = 0.71 *n*_veh_ = 19, *n*_AM11095_ = 20), 2nd (veh 59.5 ± 4.4 vs AM11095 52.4 ± 5.2, unpaired *t* test: *t* = 1.03, *p* = 0.30, *n*_veh_ = 19, *n*_AM11095_ = 20), and 3rd phase (veh 53.7 ± 6.6 vs AM11095 50.5 ± 5.9, unpaired *t* test: *t* = 0.36, *p* = 0.72, *n*_veh_ = 19, *n*_AM11095_ = 20), and morphine-dependent reduction of firing rate (Fig. 3c; veh basal 2.4 ± 0.3 Hz, veh morphine 1.6 ± 0.3 Hz, AM11095 basal 2.5 ± 0.3 Hz, AM11095 morphine 1.4 ± 0.3 Hz; two-way RM ANOVA, pre-treatment effect: *F*_(1,14)_ = 0.23, *p* = 0.88, *n*_veh_ = 9, *n*_AM11095_ = 7). On the other hand, AM11095 significantly increased the effect of morphine on the reduction of the late response to FS when compared to the vehicle (Fig. 3d; veh 44.2 ± 6.0% vs AM11095 23.1 ± 5.3%; unpaired *t* test: *t* = 2.50, *p* = 0.03, *n*_veh_ = 7, *n*_AM11095_ = 5).

### AM11095 Delayed the Onset of Tolerance to Morphine Antinociceptive Effect

Multiple studies indicated that when PEA was given as a supplement to morphine in a sub-chronic treatment, it delayed the development of tolerance to the antinociceptive effect [13, 14]. Thus, we examined whether the NAAA inhibitor AM11095 negatively modulates the development of morphine antinociceptive tolerance.

**Fig. 4:**
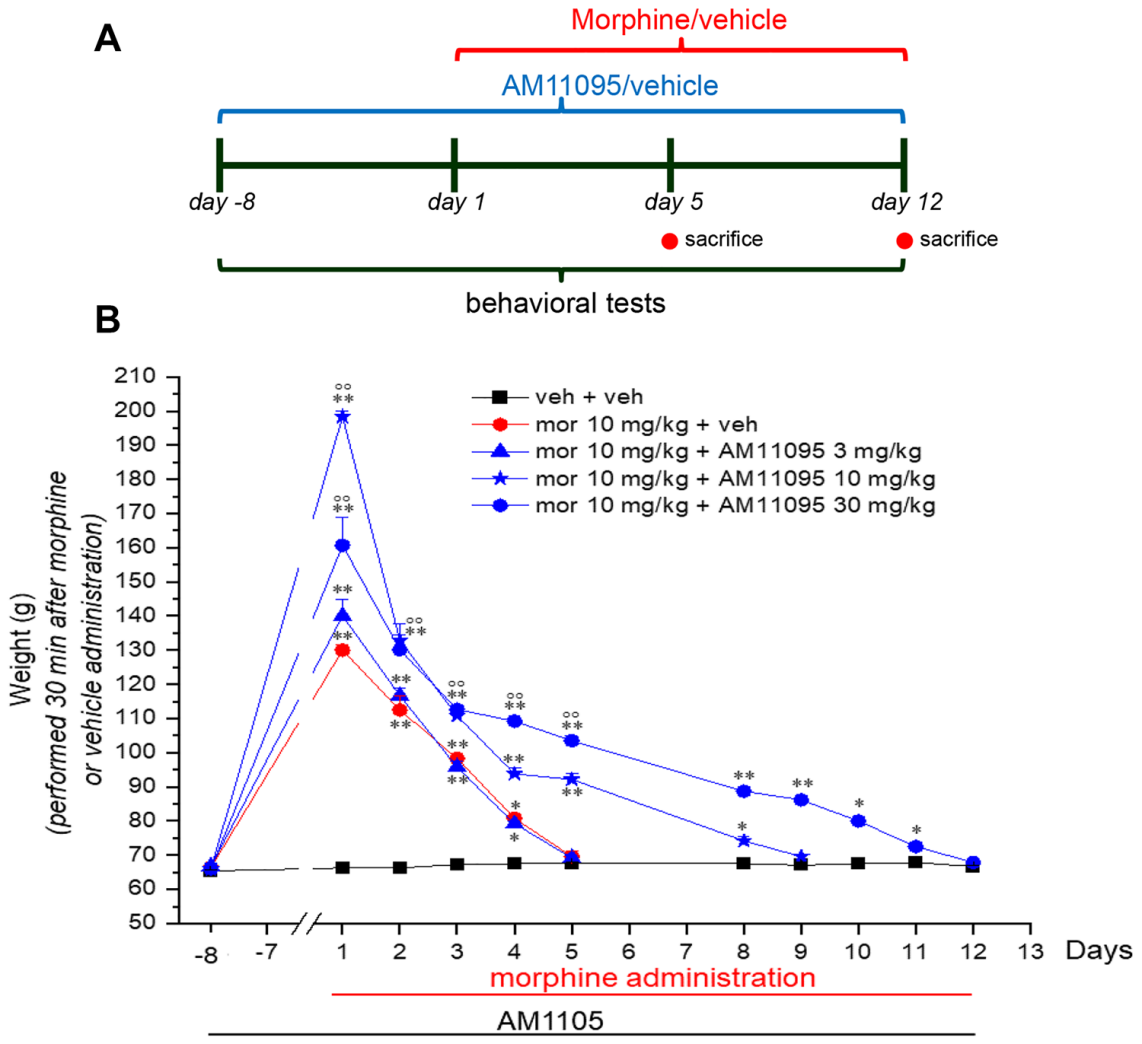
Delay of morphine tolerance development by AM11095 treatment. (**A**) Graphical depiction of the treatment protocol: treatment with AM11095 (3, 10, and 30 mg/kg daily) started 8 days before the first morphine (10 mg/kg) injection and continued during all the experiments. Behavioral measurements (paw pressure test) were performed before (0 min) and after (30 min) morphine administration and always before the first administration of AM11095. (**B**) The graph shows the time course of morphine antinociception over 12 days from day 0 to day 12 in rats pretreated with vehicle or different doses of AM11095. Data are expressed as mean ± SEM of 5 rats per group. *p < 0.05 and **p < 0.01 vs vehicle + vehicle, 30 min; °°*p* < 0.01 vs morphine + vehicle, 30 min

AM11095 was administered daily at 3, 10, and 30 mg/kg per o.s. Treatments started on day −8 to perform a pretreatment and continued in the following days until the end of the experiment. On day 1, morphine (10 mg/kg s.c.) was administered acutely and the nociceptive threshold was measured 30 min after injection by the paw pressure test (Fig. 4a). As previously described, we observed a higher antinociceptive effect of morphine in animals treated with AM11095 (10 and 30 mg/kg, Fig. 4b). Moreover, we observed a decrease in morphine effect reaching the complete lack of effect on day 5 in the morphine + vehicle group. On the contrary, the groups receiving morphine + AM11095 10 or 30 mg/kg continued to respond to morphine up to day 8, 10, or 11, respectively (Fig. 4b). This effect was dependent on the dosage of AM11095 received.

Similar results were obtained when we administered (following the same protocol) 15 mg/kg AM11095 p.o. or i.p. once per day (Supplementary Fig. 1).

### AM11095 Delayed the Onset of Tolerance to Morphine Effects on Foot-Shock-Evoked Responses

**Fig. 5:**
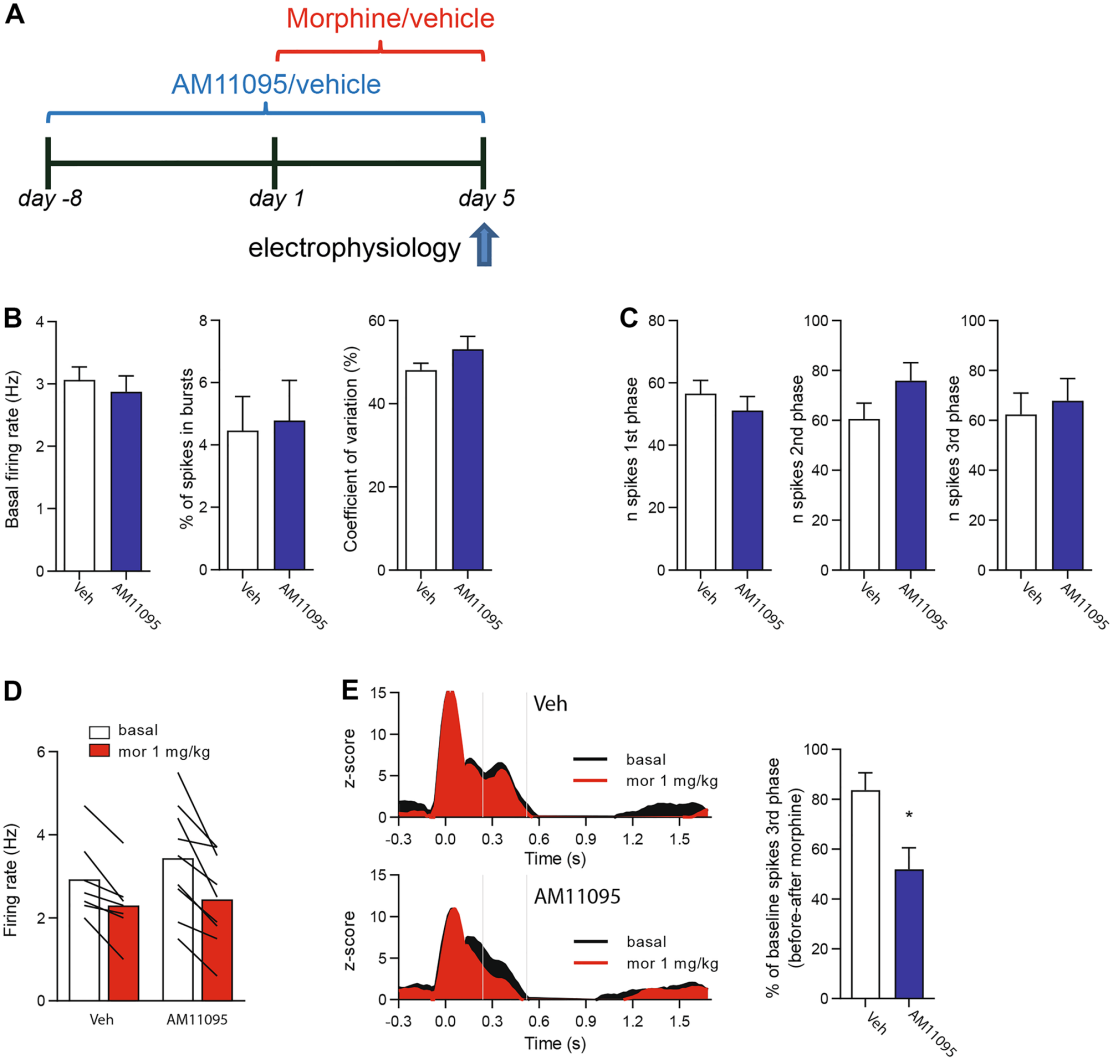
AM11095 attenuates the development of tolerance to morphine in locus coeruleus (LC) neurons. (**A**) Graphical depiction of the treatment protocol. (**B**) Bar graph representing the electrophysiological properties of LC neurons following 5-day treatment with morphine + vehicle or morphine + AM11095. Left: firing rate, center: burst firing, right: coefficient of variation. (**C**) Bar graph representing the response to FS of LC neurons following 5-day treatment with morphine + vehicle or morphine + AM11095. Left: 1st phase, center: 2nd phase, right: 3rd phase. (**D**) Bar graph illustrating that a 5-day treatment with morphine does not change morphine-induced inhibition of firing rate in LC neurons. (**E**) Left: average PSTH of the response of LC neurons to FS before and after morphine injection before and after morphine injection in vehicle (Veh, top) and AM11095 (bottom) pre-treated rats. Right: bar graph illustrating the chronic AM11095 reinstates morphine-induced attenuation of the response to FS in LC neurons in tolerant rats. Data are expressed as mean ± SEM. *p < 0.05

In light of previous behavioral results, we performed single-unit recordings on LC NA neurons in order to assess AM11095 effects on the development of morphine tolerance. Two groups of rats received a chronic treatment with morphine (10 mg/kg s.c.) + AM11095 (15 mg/kg per o.s.) or its vehicle. The treatment with AM11095 started on day − 8 while on day 1 we started morphine treatment until day 4. On day 5, when rats fully developed tolerance to morphine’s antinociceptive effects, we carried out extracellular electrophysiological recordings (Fig. 5a). We assessed spontaneous activity of LC NA neurons and their response to FS. AM11095 treatment did not alter their electrophysiological properties (Fig. 5b): firing rate (veh 3.1 ± 0.2 Hz vs AM11095 2.9 ± 0.2 Hz, unpaired *t* test: *t* = 0.57, *p* = 0.57, *n*_veh_ = 32, *n*_AM11095_ = 27), burst firing (veh 4.5 ± 1.9% vs AM11095 4.8 ± 1.3%, Mann–Whitney test: *U* = 368.5, *p* = 0.33, *n*_veh_ = 32, *n*_AM11095_ = 27), and coefficient of variation (veh 48.1 ± 1.7% vs AM11095 53.1 ± 3.1%, Mann–Whitney test: *U* = 349, *p* = 0.21, *n*_veh_ = 32, *n*_AM11095_ = 27), nor their response to FS (Fig. 5c) at the 1st (veh 56.6 ± 4.8 vs AM11095 51.2 ± 4.5, unpaired *t* test: *t* = 0.89, *p* = 0.38, *n*_veh_ = 20, *n*_AM11095_ = 19), 2nd (veh 60.5 ± 6.4 vs AM11095 76.0 ± 7.1, unpaired *t* test: *t* = 1.62, *p* = 0.11, *n*_veh_ = 20, *n*_AM11095_ = 19), and 3rd phase (veh 62.3 ± 8.7 vs AM11095 67.8 ± 8.9, unpaired *t* test: *t* = 0.45, *p* = 0.66, *n*_veh_ = 20, *n*_AM11095_ = 19) when compared to the control group. Similarly, when assessing the response to morphine (1 mg/kg i.v.), we did not detect any difference in the reduction of their firing rate between the two experimental groups (Fig. 5d; veh basal 2.9 ± 0.3 Hz, veh morphine 2.3 ± 0.3 Hz, AM11095 basal 3.4 ± 0.4 Hz, AM11095 morphine 2.4 ± 0.4 Hz; two-way RM ANOVA, pre-treatment effect: *F*_(1,14)_ = 0.38, *p* = 0.55, *n*_veh_ = 7, *n*_AM11095_ = 9). On the other hand, in line with behavioral experiments, AM11095 chronic treatment preserved morphine-induced reduction of LC NA neuron responses to FS in the 3rd phase (Fig. 5e; veh 83.7 ± 7.0% vs AM11095 51.8 ± 8.7%; unpaired *t* test: *t* = 2.73, *p* = 0.01, *n*_veh_ = 7, *n*_AM11095_ = 9). These results suggest that the NAAA inhibitor counteracted the development of morphine tolerance to the antinociceptive effects.

### AM11095 Prevented Morphine-Induced Glial Activation in the Spinal Cord

**Fig. 6:**
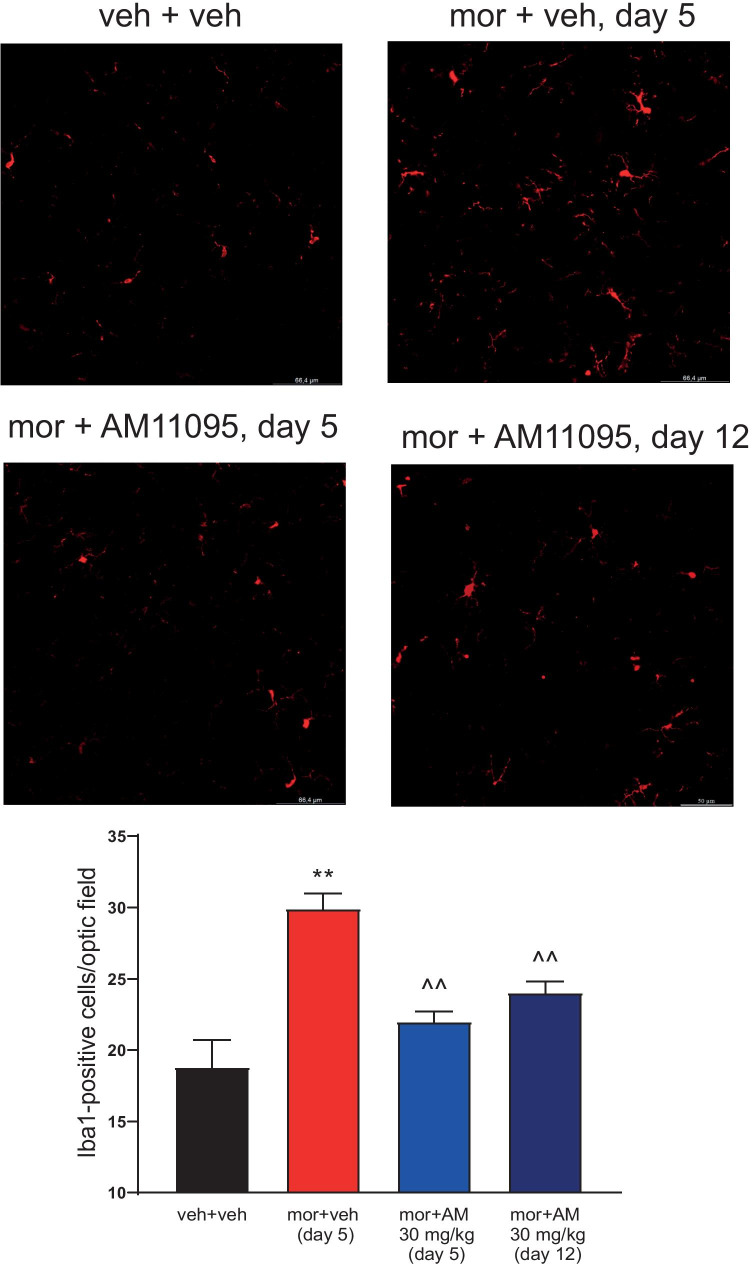
Iba1-positive cell density in the dorsal horn of the spinal cord. Treatment with AM11095 (30 mg/kg daily) started 8 days before the first morphine (10 mg/kg) injection and continued during all the experiments. Immunohistochemical analysis was performed on day 5 (morphine + vehicle-tolerant animals and morphine + AM11095 non-tolerant animals) and on day 12 (morphine + AM11095-tolerant animals) (see Fig. 4a). The number of Iba1-positive cells was measured in the dorsal horn of the L4–L5 spinal cord. Transverse sections of the spinal cord imaged with 40 × objective (scale bar = 50 μm). Histograms show the quantitative analysis reported as the number of GFAP-positive cells. Each value represents the mean of 5 rats performed in two different experimental sets. Data are shown as mean ± SEM. **p < 0.01 vs vehicle + vehicle. ^^*p* < 0.01 vs morphine + vehicle

After behavioral tests, the dorsal horn of the lumbar spinal cord was analyzed in four different groups of rats treated at different regimens: (i) vehicle + vehicle; (ii) morphine + vehicle at day 5, when tolerance to morphine was established; (iii) morphine + AM11095 30 mg/kg p.o. at day 5, when tolerance to morphine has not developed yet; and (iv) morphine + AM11095 at day 12, when tolerance to morphine has developed (Fig. 4a). On day 5, microglia density (number of Iba1-positive cells) was significantly higher in the morphine + vehicle group than in control animals (Fig. 6). On the other hand, AM11095 treatment prevented morphine-induced microglial activation. Notably, this protective effect was also maintained on day 12 (Fig. 6).

**Fig. 7:**
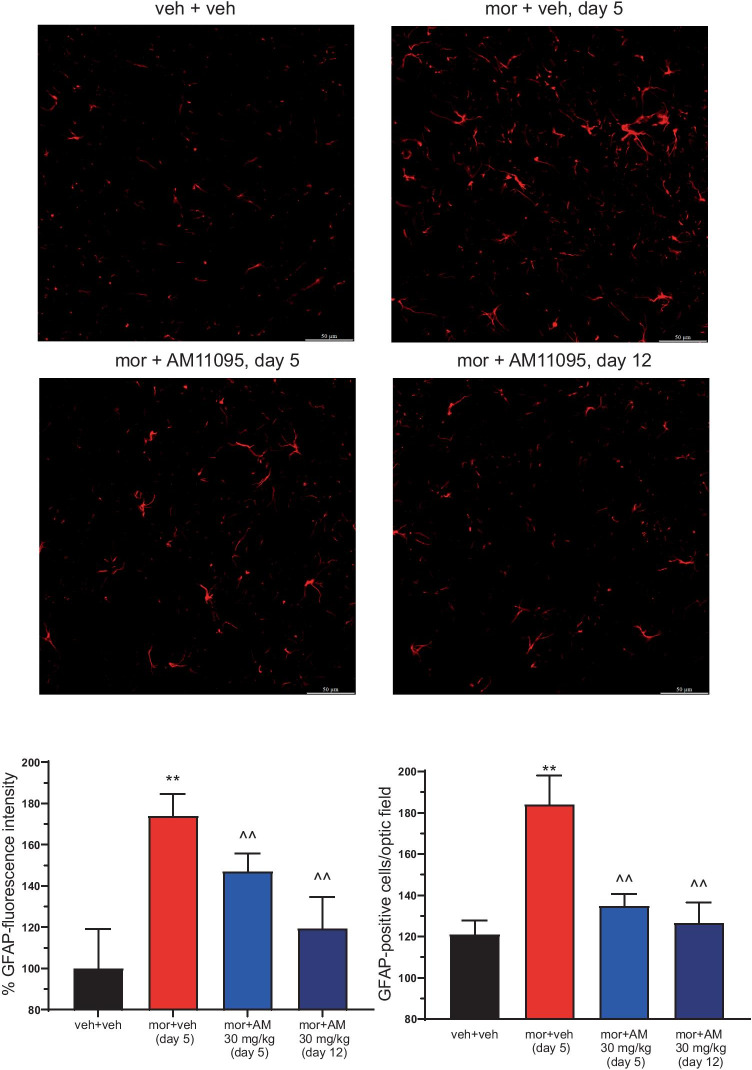
Astrocytic profile in the dorsal horn of the spinal cord. Treatment with AM11095 (30 mg/kg daily) started 8 days before the first morphine (10 mg/kg) injection and continued during all the experiments. Immunohistochemical analysis was performed on day 5 (morphine + vehicle-tolerant animals and morphine + AM11095 non-tolerant animals) and on day 12 (morphine + AM11095-tolerant animals). The number of GFAP-positive cells was measured in the dorsal horn of the L4–L5 spinal cord. Transverse sections of the spinal cord imaged with 40 × objective (scale bar = 50 μm). Histograms show the quantitative analysis reported as the number of GFAP-positive cells and % GFAP fluorescence intensity. Each value represents the mean of 5 rats performed in two different experimental sets. Data are shown as mean ± SEM. **p < 0.01 vs vehicle + vehicle. ^^*p* < 0.01 vs morphine + vehicle

The same area of the spinal cord was analyzed to study the pattern of astrocyte activation of staining cells with an anti-GFAP antibody. AM11095 prevented the maladaptive plasticity induced by morphine repeated treatment evaluated as the number of GFAP-positive cells as well as GFAP fluorescence intensity. AM11095 effect was similar both on days 5 and 12 (Fig. 7). Moreover, to deeply analyze the morphological arrangements induced by morphine and the protective effects of AM11095 treatment, a morphometric analysis of astrocytes was performed as reported in Table 1. In particular, we assessed the number of processes, the process length, and the number of connections. Morphine repeated treatment significantly increased the number of total and secondary processes and their length compared to control animals. Moreover, the number of process connections doubled in morphine-tolerant rats vs the control group. These phenomena were prevented by AM11095 as it hindered the changes in astrocyte morphology in both non-tolerant (day 5) and tolerant (day 12) animals.

**Table 1:** Morphometric analysis of astrocytes. Morphine (10 mg/kg) was dissolved in saline solution and daily subcutaneously (s.c.) administered. AM11095 30 mg/kg (split into two daily treatments of 15 mg/kg) was suspended in 1% CMC and administered p.o. starting 8 days before the first morphine injection and continued during all the experiment. Morphometric analysis of astrocytes was performed on day 5 (morphine + vehicle-tolerant animals and morphine + AM11095 non-tolerant animals) and on day 12 (morphine + AM11095-tolerant animals). Data are shown as mean ± SEM. **p < 0.01 vs vehicle + vehicle. ^*p* < 0.05 and ^^*p* < 0.01 vs morphine + vehicle

	**Treatments**			
**Parameters**	Vehicle + vehicle	Morphine + vehicle (day 5)	Morphine + AM11095 (day 5)	Morphine + AM11095 (day 12)
*n*. total processes	15.7 ± 1.6	26.8 ± 3.1*	18.4 ± 0.7^	17.3 ± 0.6^^
*n*. primary processes	4.5 ± 2.3	4.9 ± 0.2	3.9 ± 0.1	4.8 ± 0.1
*n*. secondary processes	11.7 ± 2.9	21.9 ± 3.1**	15.5 ± 0.8^	12.6 ± 0.6^^
Tot. process length (µm)	95.6 ± 9.1	166.8 ± 12.4**	111.0 ± 15.6^	115.3 ± 6.2^
Primary process length (µm)	36.3 ± 7.8	37.8 ± 3.9	29.2 ± 1.9	42.3 ± 2.5
Secondary process length (µm)	68.5 ± 14.7	129.1 ± 11.5*	81.8 ± 1.7^	73.6 ± 1.1^
Average primary process length (µm)	9.6 ± 1.2	8.8 ± 0.4	7.5 ± 0.6	8.8 ± 0.4
Average secondary process length (µm)	5.1 ± 0.7	6.3 ± 0.1	7.1 ± 1.4	6.3 ± 0.2
*n*. total connections	20.8 ± 3.4	41.4 ± 5.3*	26.7 ± 1.7^	24.5 ± 1.0^

## Discussion

Here, we show that inhibition of NAAA with AM11095 dose-dependently enhanced antinociception induced by acute morphine and delayed the development of tolerance to antinociceptive effects of morphine in rats. Investigation on the neuronal correlates of these actions revealed that AM11095 potentiated morphine-mediated inhibition of LC NA neuron response to a noxious stimulus (FS). Moreover, whereas LC NA cells recorded from animals treated with vehicle and morphine became tolerant after 5 days, a sub-chronic treatment of AM11095 in combination with morphine preserved the inhibitory effect of morphine on the response to FS. We also observed that AM11095 attenuated chronic morphine-induced activation of microglia and astrocytes in the spinal horn, contributing to the development of tolerance to opioid antinociception.

NAAA is one of the major hydrolyzing enzymes for NAEs, specifically for PEA [16, 17]. Inhibition of NAAA is one strategy to enhance endogenous levels of this lipidic neuromodulator, known to act as an endogenous agonist at peroxisome proliferator–activated receptors alpha (PPARα). Previous studies confirm that NAAA inhibition induces increases in PEA levels in specific brain regions [18] or the periphery [20, 21]. Antinociceptive and anti-inflammatory properties of PEA might be helpful against the development of opioid tolerance. The efficacy of combination therapy of opioids with PEA has been explored in preclinical behavioral investigations [13, 14]. For example, it has been shown that PEA potentiates the antinociceptive effects of morphine and delays the development of tolerance in rats [13]. Consistently, we observed that treatment with a NAAA inhibitor achieves comparable results, suggesting that endogenous PEA, similarly to the exogenously administered compound, potentiates morphine analgesia and delays tolerance.

As the primary source of NA in the CNS, the LC has been studied in numerous pain conditions, primarily due to its strategic location and network. In fact, besides the descending LC spinal pathway, critical for pain control, an ascending pathway passing through this structure appears to be responsible for the noradrenergic inputs to higher pain processing centers, such as the limbic system and frontal cortices [31]. LC NA neurons express a high density of µ opioid receptors (MOR) [32, 33] and respond to noxious stimuli, such as paw pinch and FS even in anesthetized animals [29, 34], and this response represents a qualitative fingerprint of these cells [26, 29, 35].

Electrophysiological studies carried out by Hirata and Aston-Jones (1994; 1996) [29, 30] have analyzed the LC neuron response to FS, its origin, and pharmacology. We replicated their finding of a long-latency response to FS, also called late response, which results from activation of nociceptive C-fibers in the sciatic nerve [30]. This late response is attenuated by morphine.

We found that acute inhibition of NAAA did not alter the spontaneous properties of LC but potentiated morphine-mediated inhibition of their late response to FS. Moreover, when AM11095 was co-administered with morphine during the 5-day treatment, it delayed the development of tolerance to the antinociceptive effects of morphine, without influencing the basal properties of LC NA neurons. These results are consistent with our acute and chronic analgesia experiment in awake animals. From these findings, it can be hypothesized that the activation of the neural pain circuit and chronic opioid treatment might trigger PEA signalling, which is enhanced by AM11095. Consistent with this scenario, tolerance to the antinociceptive effects of morphine is associated with increases in PEA-producing enzyme N-acyl-phosphatidylethanolamine PLD (NAPE-PLD) and PEA receptors PPARα in the spinal cord [36]. Inhibition by URB597 of the fatty acid amid hydrolase (FAAH), the other major hydrolyzing enzyme for PEA and the endocannabinoid anandamide, was shown to prevent and reverse tolerance to the antinociceptive effects of morphine in mice [36]. Together with the finding that URB597 effects were partially reversed by the PPARα antagonist GW6471 [36], these data support the hypothesis that PEA signalling via PPARα is involved in the modulation of morphine tolerance.

Indeed, preclinical and clinical evidence supports a potential role for PEA as an anti-hyperalgesic compound that might be recruited as an endogenous antinociceptive and anti-inflammatory mediator. For example, tissue levels of PEA are increased on-demand in brain areas involved in nociception and in the spinal cord following neuropathic pain induction, in human diseases associated with pain [11, 12], as well as in settings associated with injury to nervous tissue [8, 10, 37].

One hypothesis for the effects of the NAAA inhibitor on morphine acute effects and tolerance is that PEA signalling might regulate intracellular pathways downstream of MOR activation. It is plausible that NAAA inhibition might, at least partially by PPARα-mediated genomic or non-genomic effects, influence MOR density, its interaction with G-proteins, or also desensitization-initiating proteins such as GSKs, β-arrestins, or PKA. Hence, one possible mechanism to explain the potentiation of acute morphine antinociception as seen in our behavioral and electrophysiological experiments is that PPARα activation by endogenous PEA might change the ratio between phosphorylated/dephosphorylated MOR by regulating the activity of protein kinases, in a fashion similar to that already described with the β2 subunit of the nicotinic acetylcholine receptors [38–40]. Hence, MORs are constitutively phosphorylated at specific amino acids also in the absence of agonist activation [41] and ligand-induced or constitutive phosphorylation is a key process that regulates MOR acute activation and G-protein coupling efficacy, desensitization, internalization, and recycling [4].

Intriguingly, among other mechanisms potentially responsible for tolerance to chronic opioids, their properties to activate microglia and mast cells have attracted attention. Indeed, chronic exposure to opioids causes activation of these non-neuronal immune cell populations, thus contributing to an exacerbation of pro-inflammatory and pro-nociceptive processes and promoting, in the long-term, opioid-induced hyperalgesia and tolerance [42]. We confirm previous findings that chronic morphine treatment induces microglia and astrocyte activation in the dorsal horn of the lumbar spinal cord. Thus, attenuating this opioid-induced inflammatory state might prevent the development of morphine tolerance. Different groups have proposed to co-administer non-steroidal anti-inflammatory drugs or other molecules with anti-inflammatory properties and this approach revealed promising effects [42]. Among these molecules, preclinical studies demonstrate the ability of PEA to reduce inflammation and nociception induced by various acute stimuli [42] and, specifically, morphine-induced inflammation mediated by activation of non-neuronal cells [13].

Our results are in agreement with previous studies and suggest that augmentation of PEA levels by inhibition of its primary degrading enzyme NAAA prevents glial activation and might contribute to the attenuation of morphine tolerance. Interestingly, inflammatory processes such as the experimental autoimmune encephalitis in mice, a model of multiple sclerosis, are accompanied by induction of NAAA expression in the spinal cord [37] and genetic deletion of NAAA attenuates symptom intensity. This suggests that NAAA is involved in inflammatory neuropathology in different experimental settings, and reducing its function either by genetic ablation or with pharmacological tools, alleviates the severity of the pathological process.

It must be pointed out that one limitation of our study is that we have examined the effect of AM11095 on a single pain modality (paw pressure) and time point (30 min). Further studies are necessary to fully characterize the potential antinociceptive effects of NAAA inhibitors on different pain modalities (i.e., inflammatory pain).

We cannot exclude that AM11095 might interfere with morphine pharmacokinetics. AM11095 shows limited interaction with several isoenzymes of cytochromes P450 (CYP) [22], although it is not known if it interferes with UDP-glucoronosyltransferases, the main metabolizing enzymes of morphine that lead to the formation of the major active metabolite morphine-6-glucuronide. However, we tend to exclude this scenario, as the reduction of microglia and astrocyte activation in the dorsal horn induced by AM11095 when administered with morphine is not fully compatible with the hypothesis of an enhancement of morphine effects by a pharmacokinetic interaction.

Although future studies are needed to dissect the precise mechanisms by which inhibition of NAAA modulates morphine’s effect, NAAA inhibition might represent a potential novel therapeutic approach to increase the analgesic effects of opioids and to delay the development of tolerance.

## Supplementary Information

Below is the link to the electronic supplementary material.

Supplementary file1 (DOCX 285 KB)

Supplementary file2 (PDF 480 KB)

Supplementary file3 (PDF 483 KB)

Supplementary file4 (PDF 484 KB)

Supplementary file5 (PDF 484 KB)

Supplementary file6 (PDF 483 KB)

Supplementary file7 (PDF 486 KB)

Supplementary file8 (PDF 482 KB)

Supplementary file9 (PDF 483 KB)

Supplementary file10 (PDF 484 KB)

Supplementary file11 (PDF 483 KB)

## Data Availability

The data that support the findings of this study are available from the corresponding author upon request.
