# Supplementary material for: N-Acylethanolamine Acid Amidase Inhibition Potentiates Morphine Analgesia and Delays the Development of Tolerance
Source: Neurotherapeutics. 2021 Sep 22;18(4):2722–36. doi: 10.1007/s13311-021-01116-4 (PMC8804012; doi:10.1007/s13311-021-01116-4)
Supplement: Supplementary file 1 — Supplementary file1 (DOCX 285 KB) [file 13311_2021_1116_MOESM1_ESM.docx]

**Supplementary Figure S1.**

**Delay of morphine tolerance development by AM11095 per os and ip treatments.** Morphine (10 mg/kg) was dissolved in saline solution and daily subcutaneously (s.c.) administered. AM11095 was administered at 15 mg/kg daily by A) per os or B) intraperitoneally. Treatments with AM11095 started 8 days before the first morphine injection and continued during all the experiment. Behavioral measurements (Paw pressure test) were performed before (0 min) and after (30 min) morphine administration and always before the first administration of AM11095. Data are expressed as mean ± sem of 5 rats per group. *P<0.05 and **P<0.01 vs vehicle + vehicle, 30 min; °P<0.05 and °°P<0.01 vs morphine + vehicle, 30 min.
